# The Threshold Bootstrap Clustering: A New Approach to Find Families or Transmission Clusters within Molecular Quasispecies

**DOI:** 10.1371/journal.pone.0013619

**Published:** 2010-10-25

**Authors:** Mattia C. F. Prosperi, Andrea De Luca, Simona Di Giambenedetto, Laura Bracciale, Massimiliano Fabbiani, Roberto Cauda, Marco Salemi

**Affiliations:** 1 Infectious Diseases Clinic, Catholic University of the Sacred Heart, Rome, Italy; 2 Department of Pathology, Immunology and Laboratory Medicine, College of Medicine, University of Florida, Gainesville, Florida, United States of America; 3 Emerging Pathogens Institute, University of Florida, Gainesville, Florida, United States of America; 4 Infectious Diseases Unit II, University Hospital of Siena, Siena, Italy; BC Centre for Excellence in HIV/AIDS, Canada

## Abstract

**Background:**

Phylogenetic methods produce hierarchies of molecular species, inferring knowledge about taxonomy and evolution. However, there is not yet a consensus methodology that provides a crisp partition of taxa, desirable when considering the problem of intra/inter-patient quasispecies classification or infection transmission event identification. We introduce the threshold bootstrap clustering (TBC), a new methodology for partitioning molecular sequences, that does not require a phylogenetic tree estimation.

**Methodology/Principal Findings:**

The TBC is an incremental partition algorithm, inspired by the stochastic Chinese restaurant process, and takes advantage of resampling techniques and models of sequence evolution. TBC uses as input a multiple alignment of molecular sequences and its output is a crisp partition of the taxa into an automatically determined number of clusters. By varying initial conditions, the algorithm can produce different partitions. We describe a procedure that selects a prime partition among a set of candidate ones and calculates a measure of cluster reliability. TBC was successfully tested for the identification of type-1 human immunodeficiency and hepatitis C virus subtypes, and compared with previously established methodologies. It was also evaluated in the problem of HIV-1 intra-patient quasispecies clustering, and for transmission cluster identification, using a set of sequences from patients with known transmission event histories.

**Conclusion:**

TBC has been shown to be effective for the subtyping of HIV and HCV, and for identifying intra-patient quasispecies. To some extent, the algorithm was able also to infer clusters corresponding to events of infection transmission. The computational complexity of TBC is quadratic in the number of taxa, lower than other established methods; in addition, TBC has been enhanced with a measure of cluster reliability. The TBC can be useful to characterise molecular quasipecies in a broad context.

## Introduction

Phylogenetics is a branch of molecular biology that infers knowledge about taxonomy and evolution of species [1], [2]. Usually –but not exclusively- molecular phylogeny relies on a multiple alignment of genomic sequences (species, taxa), and a phylogenetic tree is a hierarchical clustering of taxa that are leaves of the tree. The taxa are implied to descent from a common ancestor. When the tree is rooted using an outgroup (a taxa known to be related but distant in terms of evolution from all the other species), each node represents the most recent common ancestor of the descendants. During the past forty years a plethora of methods that infer phylogenetic trees have been introduced, based on genetic distances, evolutionary parsimony, maximum-likelihood and Bayesian theory [2], [3], [4], [5], [6]. Genetic distances and phylogenetic trees can be inferred via different sequence evolution models and model selection criteria [7]. With some methodologies it is possible to reconstruct sequences at the internal nodes, called ancestral sequences, and also to estimate rate of evolution and to date speciation events. In addition, using resampling techniques, node splits of a phylogenetic tree can be given a measure of reliability [1], [2].

Among the prerogatives of the application of phylogenetic theory, one is the classification of taxa into distinct groups, such as genotyping or (sub)typing for viral strains, and another is the identification of pathogen transmission clusters in a sparse sample of a population, for instance groups of individuals that were infected from the same source (might be a viral strain) and transmitted the infection one to each other. Finally, another problem is to identify families within a viral quasispecies harbouring a single individual.

By cutting a phylogenetic tree at some level(s), it is possible to induce a partition of the taxa and define clusters, identifying thus non-overlapping groups of taxa or transmission events. However, the procedures for selecting optimal phylogenetic tree cut points have not been widely explored. The state-of-the-art method is a heuristic procedure that examines inter-cluster and intra-cluster distance distributions and gives a partition of the set of taxa in a phylogenetic tree, by considering the patristic distance matrix, implemented in a software named CTree [8]. This algorithm has a drawback in its complexity, which is cubic in the number of taxa. CTree has been successfully validated with the classification of type-1 human immunodeficiency virus (HIV-1) group M subtypes, but would be hardly applied for the identification of transmission clusters within large phylogenetic trees (up to several thousands of taxa, whilst the maximal number of taxa allowed in CTree for automatic cluster determination is 125). In fact, recent literature that addressed the HIV-1 transmission event identification, defined a partially-overlapping set of clusters based on a thresholding of the genetic distance matrix of the viral sequences [9], [10]. The identified clusters were then confirmed by looking at the phylogenetic tree and verifying that they were together in a subtree highly supported by the resampling statistics.

This manuscript introduces a new partition technique, the threshold bootstrap clustering (TBC), to address the taxa clustering, the transmission group identification, and the intra-patient quasispecies characterisation. The TBC is an incremental algorithm [11], and it is remarkably linked with the Chinese restaurant process, previously employed both for the clustering of microarray gene expression data [12] and for haplotype identification in ultra-deep sequencing [13].

The TBC uses models of sequence evolution and performs resampling of sequence alignments, and does not require phylogenetic tree estimation. Unlike other distance-based clustering techniques, such as k-means or partition around medoids (PAM) [14], TBC automatically determines the number of clusters without additional steps (for instance the maximisation of average silhouette values, by running multiple times k-means or PAM and using different cluster sizes), although also other methods are able to infer automatically the number of clusters [15], [16]. The computational complexity of the TBC has a quadratic upper bound, lesser in one order of magnitude than the complexity of the CTree algorithm.

Finally, coupled with the TBC, we define a methodology for assessing its robustness, calculating partition likelihood and cluster reliability, which indeed is independent on the clustering techniques and can be used with any other partition method.

## Materials and Methods

### Ethics statements

Viral isolate sequences considered in this study were obtained either querying world public data bases [17], [18] or using the proprietary retrospective HIV data base of Catholic University of Sacred Heart, Rome, Italy. For the latter, patients' written informed consent has been previously obtained and all legal aspects concerning national and international privacy policies have been accomplished, along with the approval of the ethic committee of CUSH as concerns the execution of retrospective studies. Biological samples were not collected or processed in any form for this study.

### The threshold bootstrap clustering

The core of TBC method is inspired by a Chinese restaurant process (also known as Dirichlet process), a discrete-time stochastic process [19], [20], [21]. The process can be described with the metaphor of a (Chinese) restaurant with infinite tables, where customers walk in and sit down at a table. The tables are chosen according to the following random process: (a) the first customer always chooses the first table; (b) the *n* ^th^ customer chooses the first unoccupied table with probability *a/(n−1+a)*, and an occupied table with probability *c/(n−1+a)*, where *c* is the number of people sitting at that table and *a* is a scalar parameter of the process. Intuitively, each customer entering the restaurant sits at a table with probability proportional to the number of customers already sitting at it, and sits at a new table with probability proportional to *a*. Thus, customers tend to sit at most “popular” tables that become even more crowded. By this, the process has a “power law” behaviour, where a few tables attract the majority of the customers, and the parameter *a* determines how likely a customer is to sit at a new table. Usually, in real-world problems, the Chinese restaurant process is used as a prior and a Gibbs' sampler is employed [12], [13].

In the TBC the probability assigned to any particular cluster slightly depends on the cluster size itself (this is accounted indeed in the refinement step), whilst the chance for a given object to join a cluster or to form a new one depends on how much the object is “similar” to other objects in a cluster, with respect to a known distribution that describes the overall object (dis)similarity. Since we intend to cluster molecular sequences, the measure of dissimilarity can be a genetic distance calculated via a specific evolutionary model, such as the LogDet distance [22]. In addition, the TBC is run on a column-wise bootstrap sample of the original alignment, shuffling the sequence alignment order: this allows to obtain potentially different partitions when executing the TBC, using random seeds for shuffling and bootstrap (see the next section for the likelihood assessment of partitions and the cluster reliability calculation).

**Figure 1:**
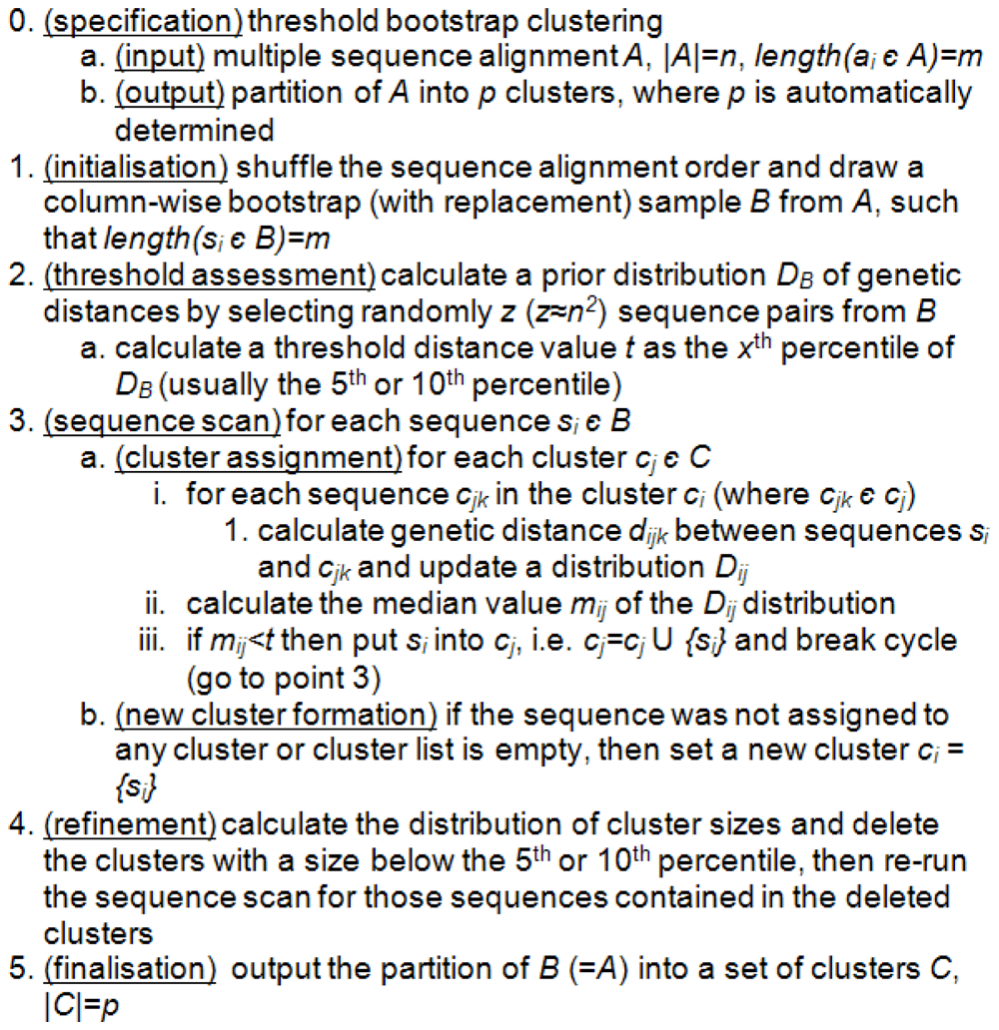
The threshold bootstrap clustering (TBC) algorithm.

The TBC algorithm starts with a multiple sequence alignment *A*, |*A*| = *n*. The algorithm initially shuffles the sequence order and draws a column-wise bootstrap sample of the alignment *B*. A preliminary phase creates an a-priori distribution *D_B_* of random pair-wise distances from *B* and calculates a threshold value *t*, corresponding to a *x* ^th^ (usually 5^th^ or 10^th^) percentile of *D_B_*. Then an empty list of clusters *C* is initialised. The sequences are scanned sequentially and the first sequence *s_1_* induces a first cluster {*s_1_*} = *c_1_ ε C*. The second sequence is compared with the first cluster and if the median value of the distance distribution obtained by comparing *s_2_* with all the elements in *c_1_* (now there is only one element in *c_1_*) is below the threshold *t*, then *s_2_* is assigned to *c_1_*, otherwise forms a new cluster. The same holds with sequence *s_3_*, which is compared with *c_1_*, and eventually with *c_2_*, if *s_2_* had formed a new cluster. Either the cluster list or the size of a cluster grows by continuing the sequence scan and distance threshold comparison. At iteration *i*, sequence *s_i_* id compared with the cluster list *C = {c_1_, …, c_k_, …, c_j_}*, where *j< = i*. The comparison starts from *k = 1* and proceeds until *j*, stopping in between if *s_i_* joins a certain cluster *c_k_*. If *s_i_* is assigned to cluster *c_k_*, *c_k_ = c_k_* U *{s_i_}*, otherwise the cluster list is incremented by a new cluster *c_j+1_ = {s_i_}*, i.e. *C = C* U*{c_j+1_}*. After each sequence has been examined (*i = n*), a post-processing phase starts. By following the Chinese restaurant dogma, for which popular clusters tend to attract single elements, we calculate a distribution of cluster sizes and we delete the clusters whose size is below the 5^th^ (or 10^th^) percentile. Then the cluster assignment phase is re-run for those sequences belonging to the deleted clusters. Finally, the number of clusters corresponds to the size of |*C*|. The TBC algorithm is explained in detail in Figure 1.

The computational complexity of the TBC is *O(n^2^)*, where *n* is the number of objects to be clustered: in fact, the threshold assessment phase requires *n^2^* random object comparisons for the estimation of *D_B_*, and the sequence scan phase in the worst cases would create either a unique cluster or *n* distinct clusters, corresponding to *n(n+1)/2* comparisons.

In order to speed up the algorithm in our implementation, we limited the number of random distances to be calculated in the interval [1000, 500000], with an additional control on the threshold *t*, calculated at every 100^th^ iteration, stopping the procedure if the difference between two consecutive estimated thresholds was below 0.0000001. In the sequence scan phase, each distribution *D_ij_* was approximated by considering a limited number of comparisons with the objects in a cluster equal to the square root of the cluster size, with a minimum of 10 comparisons (unless the cluster size was smaller) and a maximum of 100, i.e. *min{max{sqrt(*|*c_i_*|*),10},100}*.

### Assessment of partition likelihood and cluster reliability

The TBC clustering induces a full partition of the taxa objects into *p* clusters, where *p* is automatically determined. By varying the initial conditions (i.e. random seeds for taxa shuffling and sequence bootstrap), TBC can produce different partitions, both in the number of clusters and in the elements belonging to each cluster. As maximum-likelihood and Bayesian estimations are used to select both for best phylogenetic trees under a set of model parameters, and to infer node reliability, we might be interested to assess the most plausible partition(s) obtained from multiple runs of the TBC and to determine reliability of each cluster. Of note, such a methodology would apply to any clustering technique that can produce different partitions by varying its initial conditions.

By reviewing the literature, this problem has gained growing attention in the recent years, acquiring the name of “consensus” or “ensemble” clustering [23]. Consensus clustering tries to find a single partition which is a better fit under some goodness-of-fit functions with respect to other existing partitions. The consensus partition does not necessarily coincide with any of the original partitions. The cluster-based similarity partitioning algorithm, the hyper-graph partitioning algorithm, or k-means based algorithms [24] are a few of the many variations on a theme.

We propose here, differently from most of consensus clustering algorithms, a methodology that selects *one* particular partition in a set of obtained partitions.

Partitions can be compared statistically to determine their agreement, using the adjusted Rand index (ARI) [25], an indicator of cluster agreement which corrects for chance and takes values in [0,1]. By using the ARI, given a set of partitions *P*, |*P*| = *m*, we can compute the likelihood of a partition with respect to the others *p_i_ ε P* as *L(P| p_i_) = Pr(p_i_|P) = ∏_j≠i_a_ji_*, where *a_ji_* is the ARI between partition *p_j_* and *p_i_*, and then select the best partition *p^b^* with the maximum likelihood. In this case, we are assuming that the ARI is directly proportional to a probability, i.e. *a_ji_* ∝ *L(p_j_| p_i_) = Pr(p_j_|p_i_)*.

Once the best partition is determined, we estimate the reliability of each cluster with a procedure similar to the posterior probability estimation for nodes of a phylogenetic tree under Bayesian monte-carlo analysis [1]. In detail, the partitions *p_i_ ε P* are ordered decreasingly by their associated likelihood and the last *x* ^th^ percentile (usually from the 75^th^ or above) of partitions is deleted. Each retained partition *p_i_* is compared with the best partition *p^b^* and for each cluster *c^b^_i_ ε p^b^* a support value is defined as follows: (i) for each partition *p_j_ ε P*, *j≠b*, identify the cluster c^*^_jk_ ε p_j_ such as c^*^_jk_ ∩ c^b^_i_ is the maximum among all possible intersections *c_jk_ ∩ c^b^_i_*; (ii) calculate the support *s^b^_ij_* as s^b^_ij_ = c^*^_jk_ ∩ c^b^_i_/c^*^_jk_ U *c^b^_i_*. Then the overall support *s^b^_i_* for a cluster *c^b^_i_* is the average value of all *s^b^_ij_*.

### Data sets, software and settings of comparison methods

The TBC has been entirely implemented in java [26]. Procedures for likelihood and cluster reliability assessment have been written using the R mathematical software suite [27]. The whole source code is available as a supplementary material (File S1). The CTree [8] algorithm was used as a comparison method, along with the PAM (using the LogDet estimator as a distance measure) where the optimal number of clusters was assessed via the average silhouette value maximisation [14].

The TBC was tested in the following scenarios: (i) identification of HIV-1 subtypes using a standard reference set, downloaded as a pre-made alignment from the Los Alamos repositories [17], considering either full-length genomes or *pol* genes; (ii) identification of HCV subtypes, using a standard reference set, downloaded as a full-genome pre-made alignment from the Los Alamos repositories [18]; (iii) identification of HIV-1 subtypes using a larger full-genome population sample, downloaded as a pre-made alignment from the Los Alamos repositories [17]; (iv) identification of inter-patient quasispecies (discriminating among different patients) using a HIV-1 subtype B data set, downloaded as a full-genome pre-made alignment from the Los Alamos repositories [18]; (v) identification of inter/intra-patient quasispecies (discriminating within the same patients and among different patients) using a data set previously analysed by Shankarappa et al. [28], composed by HIV-1 *env* (C2-V5 region) isolates sampled from different patients, followed up from 6 to 12 years after seroconversion, until the development of advanced disease.

As a final evaluation (vi), the TBC was also applied to a set of HIV-1 group M subtype B polymerase sequences obtained from the private CUSH clinical data base, identifying viral isolates coming from patients with known transmission history (collecting any sequence at any time point). A set of control sequences was added to this data set: specifically, samples coming from other HIV positive patients followed up at CUSH, with unknown transmission history, two outgroups (HIV-1 subtypes C and J), and the reference HIV-1 subtype B HXB2 strain. Sequences were aligned using ClustalW [29]. Resistance of each viral sequence with respect to an antiretroviral class (nucleoside-tide/non-nucleoside/protease inhibitors) was defined as the presence of at least one amino-acidic mutation panelled by the International AIDS Society (any major mutation for protease) [30], by aligning pairwisely each sequence against the HIV-1 consensus B, with an in-house modified version of the local Smith-Waterman-Gotoh alignment algorithm implemented in java [31], [32]. Columns of the multiple alignment corresponding to codon positions previously associated to drug resistance were deleted, in order to avoid the possible bias coming from convergent evolution due to treatment experience.

For each TBC analysis [data sets (i) to (vi)], the distance threshold percentile was evaluated in the interval *t* = [1.0, 45] with step sizes of 0.5/0.25, and 200 bootstrap runs were executed. For the analyses of HIV/HCV subtyping (reference data sets), CTree has been executed on phylogenetic trees constructed by neighbour-joining and LogDet estimator. A patient from the data set analysed by Shankarappa et al. [28] was analysed in depth by estimating a Bayesian phylogeny using BEAST [33]. For the analysis of transmission clusters on CUSH data, a maximum-likelihood tree was estimated, setting up a general-time-reversible model, with a 20-parameter gamma optimisation, and a mix of nearest-neighbor interchanges and subtree-prune-regraft moves for tree topology search, using the FastTree software [34], [35]. Reliability of each tree split was calculated by a Shimodaira-Hasegawa test. The internal sensitivity parameter of CTree was always set to 1 (slowest as concerns computational time, but correspondent to maximal accuracy).

## Results

### HIV-1 subtyping

HIV is divided into two *types* (type-1 and 2), and into four *groups* (M, N, O, P) [36], [37]. Group M is the most widespread and is divided into several *subtypes*, lettered from A to K. Subtypes have been historically defined by a human-driven crisp clustering obtained by analysing different phylogenetic trees constructed over different HIV genes, but do not necessarily correspond to an optimal grouping under specified constraints. HIV can also recombine and a number of strains composed by mixed subtypes has been described. During the past years, the subtype nomenclature has been undergoing many revisions: for instance, subtypes E and I have been discovered to be indeed recombinant forms and removed from list of pure subtypes. However, considering the large and long-lasting research done on HIV subtyping, the current classification can be considered as reliable [36], and it is used as a standard reference for expert systems that infer subtype for patients' sequences [38].

**Figure 2:**
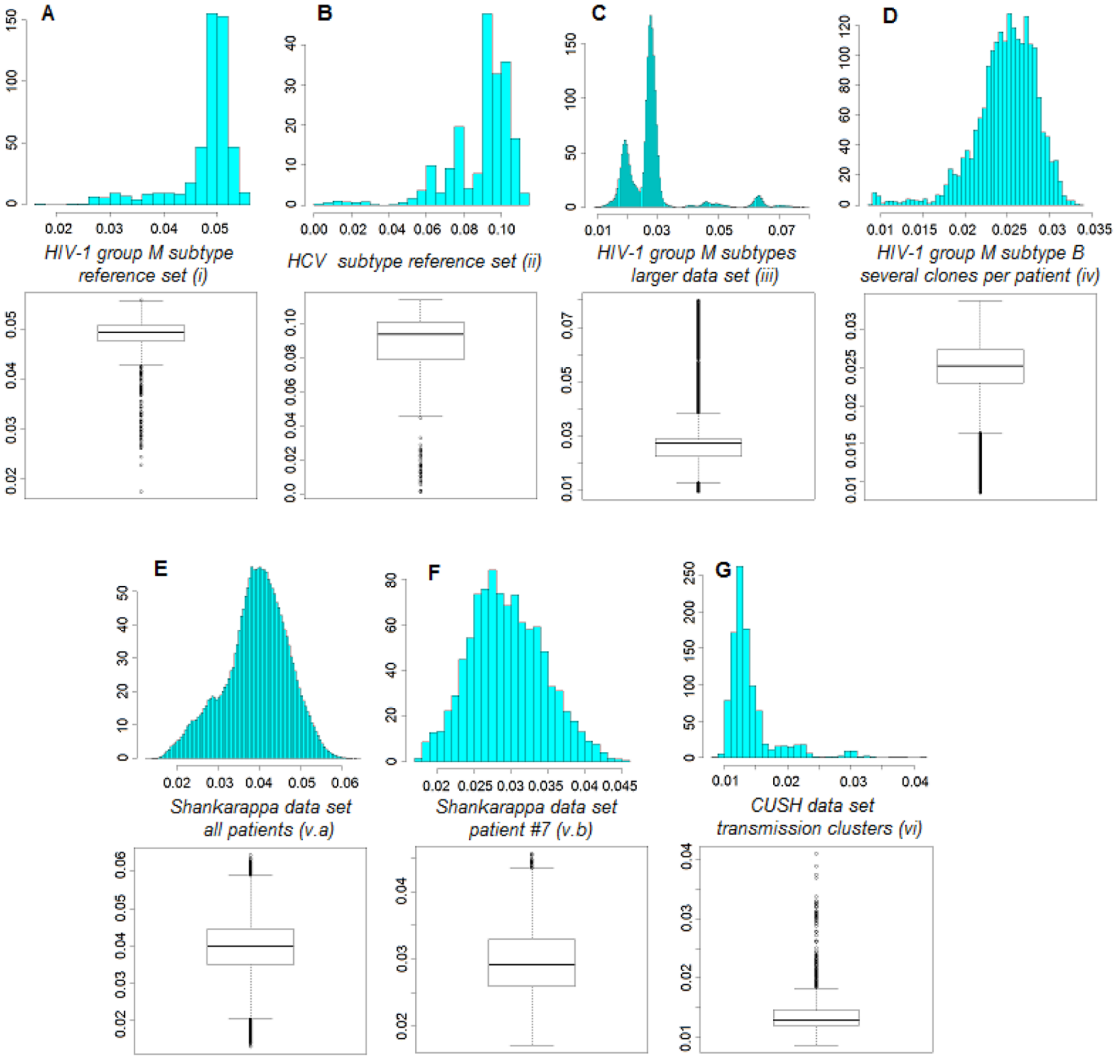
Pairwise distance distributions. Distributions of pairwise distances using the LogDet estimator for the data sets (i) – (vi) analysed in this study.

**Figure 3:**
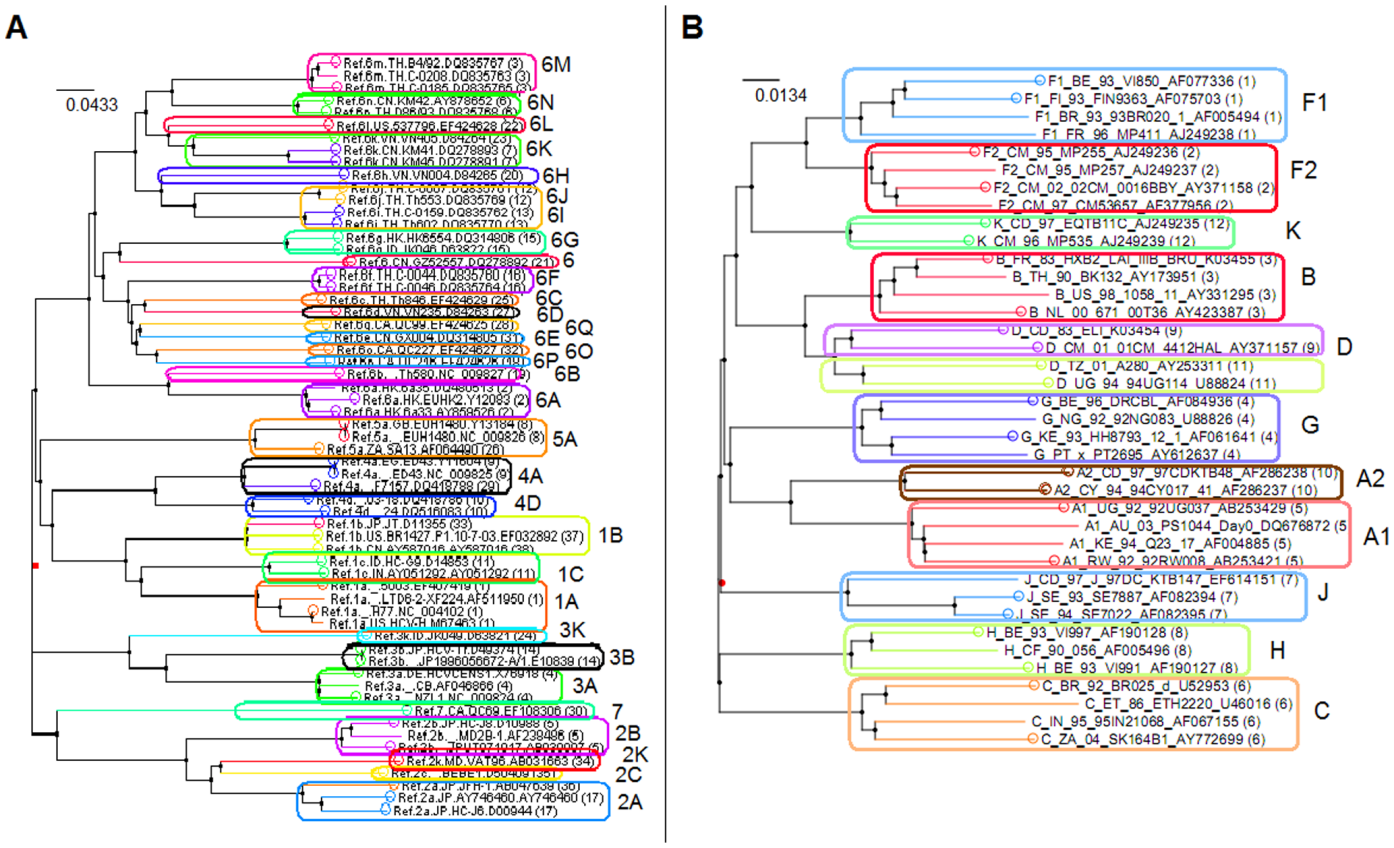
Phylogeny and TBC of HIV/HCV subtypes. Phylogenetic trees constructed for the HCV genotype (panel A) and group M HIV-1 subtype (panel B) reference sets of Los Alamos repositories (neighbour-joining, LogDet distance). Coloured branches represent clusters retrieved by the CTree algorithm, whilst circles represent clusters retrieved by the TBC algorithm.

The HIV subtype reference multiple alignment (i) was composed by 38 representative sequences of 11 pure subtypes (on average 3.4 sequences for each subtype). TBC was run either on the whole genome (≈9,000 bases) or restricting on the polymerase gene (≈2700 bases), which is the routinely sequenced gene in clinical practice for drug-resistance testing. By considering the full-length genome set, a TBC tuned on *t* = 10 yielded a perfect concordance with the HIV subtypes, producing exactly 11 clusters, with an ARI between clusters and real subtypes of 1.0, a median (IQR) cluster support of 95% (72%–100%). Figure 2, panel A, depicts the distribution of pairwise distances, using the LogDet estimator, with a median (IQR) distance of 0.050 (0.048–0.051). The CTree also produced a perfect partition, whilst the PAM with silhouette maximisation identified exactly all subtypes except for B and D, that were pooled together. When executing the TBC using the sole *pol* gene, at *t* = 10 all sequences but one were partitioned correctly (Figure 3, panel B): ARI was 0.967, median (IQR) cluster support was 92% (63%–98%). The misplaced sequence was a subtype D, that clustered alone (support was 42%). By looking at the whole set of partitions, often subtype D divided into two distinct clusters (formed either by one or two sequences out of four). The CTree algorithm yielded an ARI of 0.955; in this case two subtype D sequences formed a cluster apart, similarly to the TBC. The PAM, instead, pooled together subtype B and D. As expected, the number of clusters decreased by increasing the percentile threshold: in detail, for the whole genome case, at *t* = 5 ARI was 0.511 and number of clusters was 26, whilst at *t* = 25 ARI was 0.375 and number of clusters was 6.

A larger set of full-length genomes HIV-1 isolates with known subtype was also considered (iii). The data set was composed by 1,258 isolates, with a median (IQR) number of 19 (4–63) isolates per subtype. There was exactly one sequence per patient, and the median (IQR) distance was 0.027 (0.022–0.029) (Figure 2, panel C). The optimal threshold for TBC was found at *t* = 25, obtaining an ARI of 0.99 and a median (IQR) cluster support of 97.8% (91.9%–99.7%). The CTree algorithm was not executed on this data set, because of the high number of isolates. The PAM clustering yielded an ARI of 0.894.

### HCV subtyping

As a second analysis, TBC was run on the Los Alamos HCV genotype reference set (ii). HCV nomenclature is not as well established as that of HIV. HCV has been divided into a few major *genotypes* (numbered from 1 to 7, where the word genotype should correspond ideally to the group for HIV) and several *subtypes* (lettered alphabetically). Recent studies have strongly revised HCV classification [39], and still some subtypes have to be confirmed (a confirmation of a subtype is when at least two or three whole-genome sequences coming from patients that are not epidemiologically linked cluster together under different phylogenetic analyses and do not exhibit recombination patterns). The current HCV reference set from Los Alamos comprises both confirmed and unconfirmed sequences: the downloadable whole genome (≈9,500 bases) pre-made alignment is composed by 61 distinct sequences, 7 genotypes (thus 8.7 sequences per genotype), and 32 subtypes (1.9 sequences per subtype, and 4.6 subtypes for each genotype). The median (IQR) pairwise distance was 0.094 (0.079–0.101), as shown in Figure 2, panel B. Only whole genome analysis was carried out.

In the HCV analysis we obtained the best subtype concordance at a lower percentile threshold, i.e. *t* = 4. Thirty-one clusters were identified, the ARI was 0.952, and the median (IQR) cluster support was 96% (79%–100%). Differently from the given classification, TBC put together subtypes 6i and 6j (Figure 3, panel A). The CTree yielded 38 clusters, with an ARI of 0.842: differently from the TBC output, subtypes 1b, 2a, 4a, 5a, and 6k were split into three, two, two, two, and two clusters, respectively, whilst subtypes 6i and 6j were correctly identified. The PAM found 24 clusters, with an ARI of 0.815; in this case, several genotypes were misplaced.

### HIV-1 group M subtype B inter/intra-patient quasispecies analysis

For this analysis (iv), 356 HIV-1 subtype B isolates were considered, from 83 patients, with multiple samples or clones per patient. The median (IQR) number of isolates per patient was 3 (2–4). The median (IQR) pairwise distance was 0.025 (0.023–0.027) (Figure 2, panel D. The primary objective was to identify a cluster for each population sample of a patient. The TBC found 110 clusters, with an ARI of 0.934 at the optimal threshold of *t* = 1.9. The median (IQR) cluster support was 93% (72%–100%). In this case, the PAM algorithm yielded 73 clusters and an ARI of 0.933.

### Analysis of the “Shankarappa” [28] data set

This data set (v) was composed of 1300 isolates from 9 patients, with a median (IQR) pairwise distance of 0.040 (0.035–0.044) (Figure 2, panel E). The TBC was primarily run on the whole data set, trying to cluster the different patients. At *t* = 7.5, we obtained the best ARI of 0.716 (58 clusters), with a median (IQR) cluster support of 64% (47%–74%). The PAM algorithm yielded 8 clusters and an ARI of 0.260.

**Figure 4:**
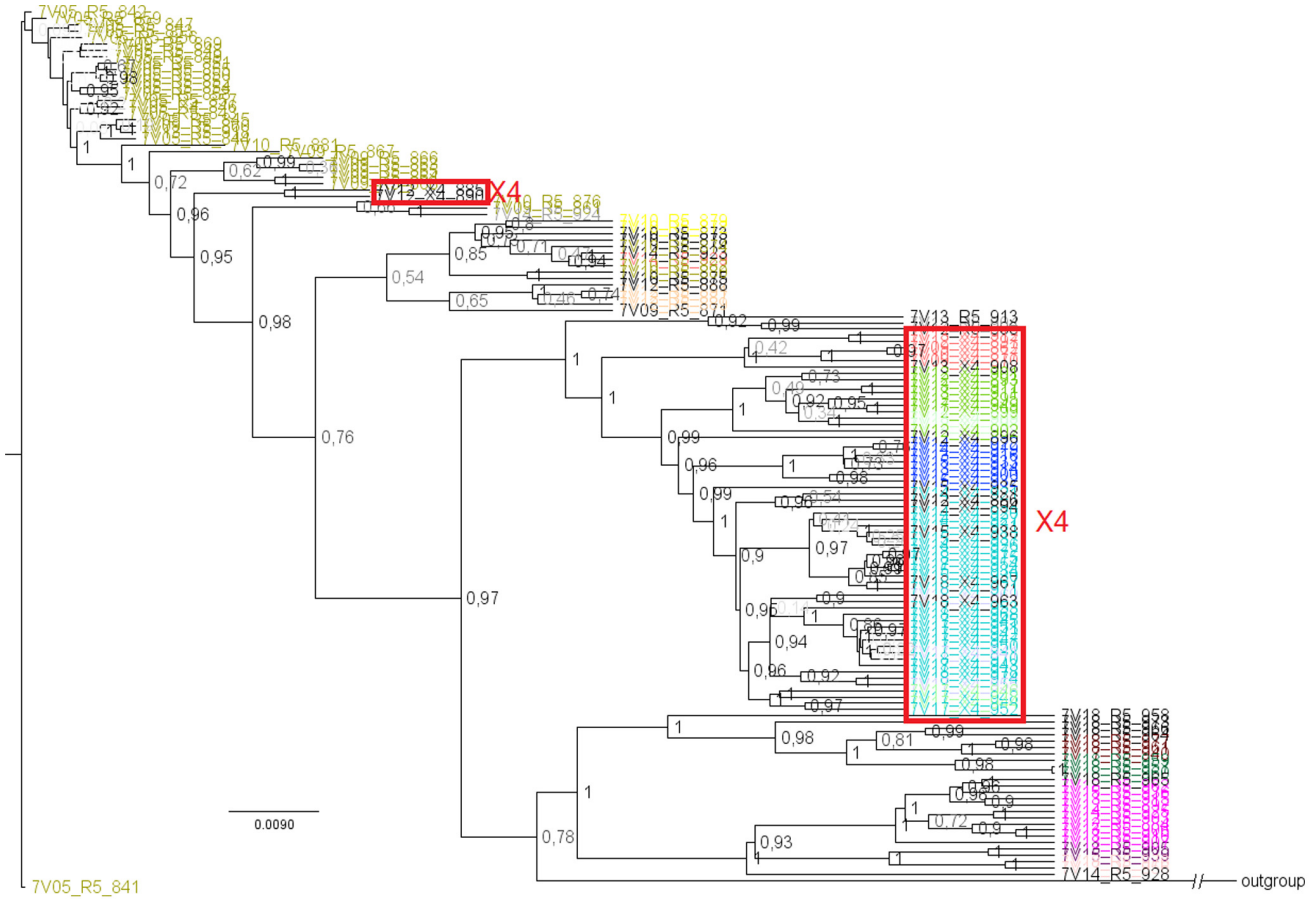
Intra-patient phylogeny and TBC. Bayesian phylogenetic tree for a particular patient (# 7) from the Shankarappa [28] data set. Tree is rooted on the earliest sequence, and node labels represent posterior probabilities. Coloured tips correspond to different clusters retrieved by the TBC using a threshold of 12 (whilst black tips are singletons). X4-tropic populations are enclosed in red-boxes.

Successively, a single patient (Figure 2, panel F) was selected from this data set (patient # 7, n = 138 sequences) and analysed separately, estimating a Bayesian phylogeny, highly resolved, depicted in Figure 4. The tree was rooted on the earliest sequence and showed a clear ladder-like topology, where the more distant branches usually corresponded to later time points, which is typical of a longitudinal HIV-1 sampling. Since there was not an already defined clustering (except for the time point indicators), we optimised the threshold by maximising the average silhouette value, as it was done for the PAM algorithm [14]. At a threshold corresponding to the 12^th^ percentile, the TBC produced a partition (39 clusters) that was clearly following the evolution through time highlighted by the phylogenetic analysis (Figure 4). In addition, the TBC identified correctly the sub-population that evolved into an X4-tropic virus.

### Transmission cluster identification

The last analysis (vi) was executed on the CUSH dataset (Figure 2, panel G), comprising HIV-1 subtype B isolates from patients with known transmission history: there were 12 known transmission events from patient-to-patient (n = 66 sequences, with 5.5 sequences per transmission event, and exactly two patients in each transmission event), 6 control patients from CUSH (12 sequences), two outgroups (subtype C and J), and the subtype B HXB2 reference isolate. The best ARI between transmission groups and clusters generated by TBC was 0.682 at *t* = 7, with a median (IQR) cluster support of 75% (56%–88%). All control sequences except one were correctly placed, whereas, when looking at the transmission events, only 3/12 (25%) transmission events were uniquely determined by placing both patients (and only those) in the same cluster (supplementary Figure S1). The TBC was able in general to identify and cluster viral isolates belonging to the same patient, but not extremely sensitive to identify transmission events, although the sample size of this experiment was small and sparse as concerns times of sampling. The CTree algorithm yielded a poorer performance, with an ARI = 0.35, identifying correctly only 2/12 (16.7%) transmission events. The PAM did not identify any transmission group, and selected only 2 clusters, with an ARI of 0.001.

As an additional comparison, we used also the method proposed in [9], [10], which can be considered the state-of-the-art with respect to HIV-1 transmission cluster identification. The procedure identified 3/12 (25%) clusters that were confirmed by node reliability values >90% from the maximum-likelihood phylogenetic tree.

However, the best method for identifying transmission clusters still remains a human interpretation of the phylogenetic tree (supplementary Figure S1). By looking at the node reliability (>90%) and (sub)tree branch lengths, we identified manually 6/12 (50%) clear transmission events. Of note, even the phylogenetic tree was not resolving correctly all the transmission events. In a few cases, two patients belonging to the same transmission event clustered apart from each other, or mixed with other events/controls. More severely, there were patients whose sequences were not even clustering always together in the tree.

## Discussion

In this manuscript we introduced the threshold bootstrap clustering, a new incremental methodology for partitioning molecular sequences. The TBC is inspired by a stochastic Chinese restaurant process and takes advantage of resampling techniques and models of sequence evolution. The TBC uses as input a multiple alignment of molecular sequences and its output is a crisp partition of the taxa into an automatically determined number of clusters. By varying initial conditions, TBC can produce different partitions.

We described also a procedure for selecting a partition among a set of candidate ones and a measure of cluster reliability. Note that our definition of the likelihood of a partition is not an absolute measure of the “goodness” of the partition, but expresses how much a partition is similar to all the generated partitions (in this sense is seen as a consensus partition) and is of use for assessing cluster reliability.

TBC was successfully tested for the identification of group M HIV-1 and HCV subtypes and compared with an established methodology, the CTree algorithm [8], and the with the PAM algorithm [14]. The CTree algorithm works on patristic distances of a phylogenetic tree (but can work on any general distance matrix) and produces a crisp partition in *O(n^3^)* complexity. The TBC is based as well on distances and runs in *O(n^2^)* complexity.

TBC was as good as CTree in identifying ex-novo the group M HIV-1 subtypes and outperformed slightly CTree in the HCV subtyping problem. Our algorithm showed also good performance in clustering larger data sets and in identifying inter/intra-patient quasispecies. It was also efficient in partitioning longitudinal intra-patient data, and could be useful in other related contexts, such as the detection of dual infections.

In principle, the TBC might be applied also to next-generation sequencing data alignments, and the sequence length should not be a serious problem if Roche 454 Life Science [40] technology is used to amplify specific regions. With shotgun sequencing the data need to be analysed via sliding windows, and then a problem of variant reconstruction arises. We do not know how the TBC would behave in presence of sequencing errors that should be -at least- corrected before running the clustering. However, another approach that uses the Chinese restaurant process as a prior to infer clusters via Gibbs' sampling has been recently proposed, and performs both clustering and error correction at the same time [13].

TBC was also evaluated in the problem of transmission event identification. Using a data set of patients followed up at the Catholic University of Sacred Heart in Rome, Italy, with known transmission history, TBC was able to identify transmission events in 25% of cases, whilst CTree assessed on 16.7%. The transmission event dataset was also evaluated using a previously published method [9], [10], specifically tuned for HIV transmission cluster identification, and that method identified 25% of transmission events. With a human-visual evaluation of subtrees and node reliability of a maximum-likelihood phylogenetic tree, we were able to infer correctly 50% of transmission events. Thus, even a detailed phylogenetic analysis was not able to resolve all transmission events. In fact, for HIV it has been shown previously that many factors (such as long period of infectivity, sparse time and space sampling) can limit the concordance of phylogenetic reconstruction and the reported epidemiological evidence [41], [42], [43]. The transmission event data set of CUSH was composed by sequence samples of patients taken at different times and disease stage: some patients were sequenced multiple times either before treatment initiation or at treatment failures, whilst others had only one sequence sample taken. We recognise that a larger and less sparse data set would be desirable in order to assess better the TBC performance on this particular problem.

This work has some limitations. First, the TBC algorithm might be sensitive to the percentile threshold, which is a free parameter. A value of 10 was optimal for the problem of group M HIV-1 subtyping, whereas lower thresholds were needed for the HCV and the subtype B HIV-1 transmission data sets (4 and 7, respectively). In our study we did not find a direct correlation between the median pairwise distance and the optimal threshold, considering also that the majority of the data sets did not present a normal distribution of distances.

A way to optimise the percentile threshold -without knowing a priori the sequence grouping- is to run the TBC using different thresholds and then calculate for each partition a cluster validity measure, such as the Goodman-Kruskal index, the Dunn's index, or the average silhouette value [14], [44], [45]. We have implemented a few of these indices in the released software, which can be used effectively to optimise the threshold. Nonetheless, this issue warrants further investigations, especially when different objectives rather than the viral subtyping are prosecuted, as it is for transmission event identification or intra-patient quasispecies grouping. It might be that the optimal threshold identified by a statistical analysis does not necessarily correspond to a suitable grouping from a biological/clinical point of view. However, for most of the cases, the optimal threshold selected by an index maximisation was concordant with the experimental results (see the supplementary Figure S2).

A second limitation is the cluster support calculation, defined in a very simple manner. In fact, in some degenerate cases can be completely uninformative, such as when all the partitions are composed by either all single clusters or a unique one, yielding always a cluster support of 100%.

Another problem that was not covered in this work was how to deal with the presence of recombinant viral strains in the data sets to be clustered. The TBC could produce unstable partitions when recombinants happen to be included, since depending on the bootstrap sampling they might jump among different clusters. This might also affect the overall cluster support calculation. A possible approach could be the relaxation of the crisp clustering, considering a soft clustering model where instances can belong to different clusters with different grades of membership.

In conclusion, the TBC has been shown to be as effective as, if not better, than previously published methods for the clustering of viral strains, under different scenarios. TBC has the advantage of a quadratic complexity, and there is the possibility to identify a consensus partition and a measure of cluster reliability. Although conceptually different and presumably with less expressional power than a full phylogenetic analysis, the TBC might be useful for the processing of large-scale sequence data sets, where both the phylogenetic software and the standard clustering algorithm might be hardly applicable.

## Supporting Information

Figure S1 Maximum likelihood tree estimated using sequences from patients with known transmission history (n = 66, with 12 transmission events) and control sequences, all collected at the Catholic University of Sacred Heart in Rome, Italy. Phylogenetic tree is rooted using HIV-1 J and C subtypes. Different colours highlight different transmission events. Coloured boxes indicate a transmission cluster uniquely determined and supported by a node reliability >90%. By visual inspection of the tree, 6/12 transmission events could be resolved. The TBC algorithm identified correctly 3/12 transmission events (indicated with coloured bullets).
(1.53 MB TIF)

Figure S2 Optimisation of the threshold value by considering a cluster validity index (in this case maximising the difference between the median inter/intra-cluster distance distributions of a partition and the median values obtained from a random partition with the same number of clusters).
(0.16 MB PNG)

File S1 Software source and compiled code
(0.02 MB ZIP)
