# Supplementary figures and images for: The Threshold Bootstrap Clustering: A New Approach to Find Families or Transmission Clusters within Molecular Quasispecies
Source: PLoS One. 2010 Oct 25;5(10):e13619. doi: 10.1371/journal.pone.0013619 (PMC2963616; doi:10.1371/journal.pone.0013619)

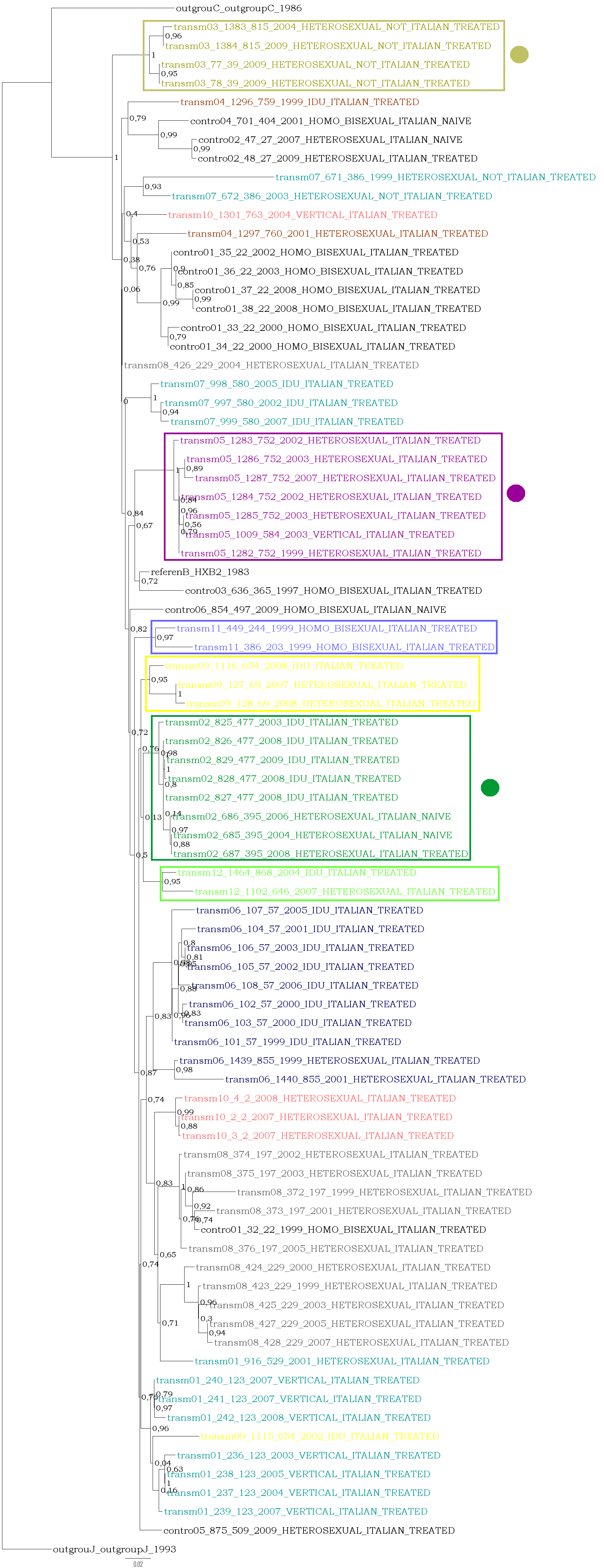

Supplement: Figure S1 — Maximum likelihood tree estimated using sequences from patients with known transmission history (n = 66, with 12 transmission events) and control sequences, all collected at the Catholic University of Sacred Heart in Rome, Italy. Phylogenetic tree is rooted using HIV-1 J and C subtypes. Different colours highlight different transmission events. Coloured boxes indicate a transmission cluster uniquely determined and supported by a node reliability >90%. By visual inspection of the tree, 6/12 transmission events could be resolved. The TBC algorithm identified correctly 3/12 transmission events (indicated with coloured bullets). (1.53 MB TIF) [file pone.0013619.s001.tif]

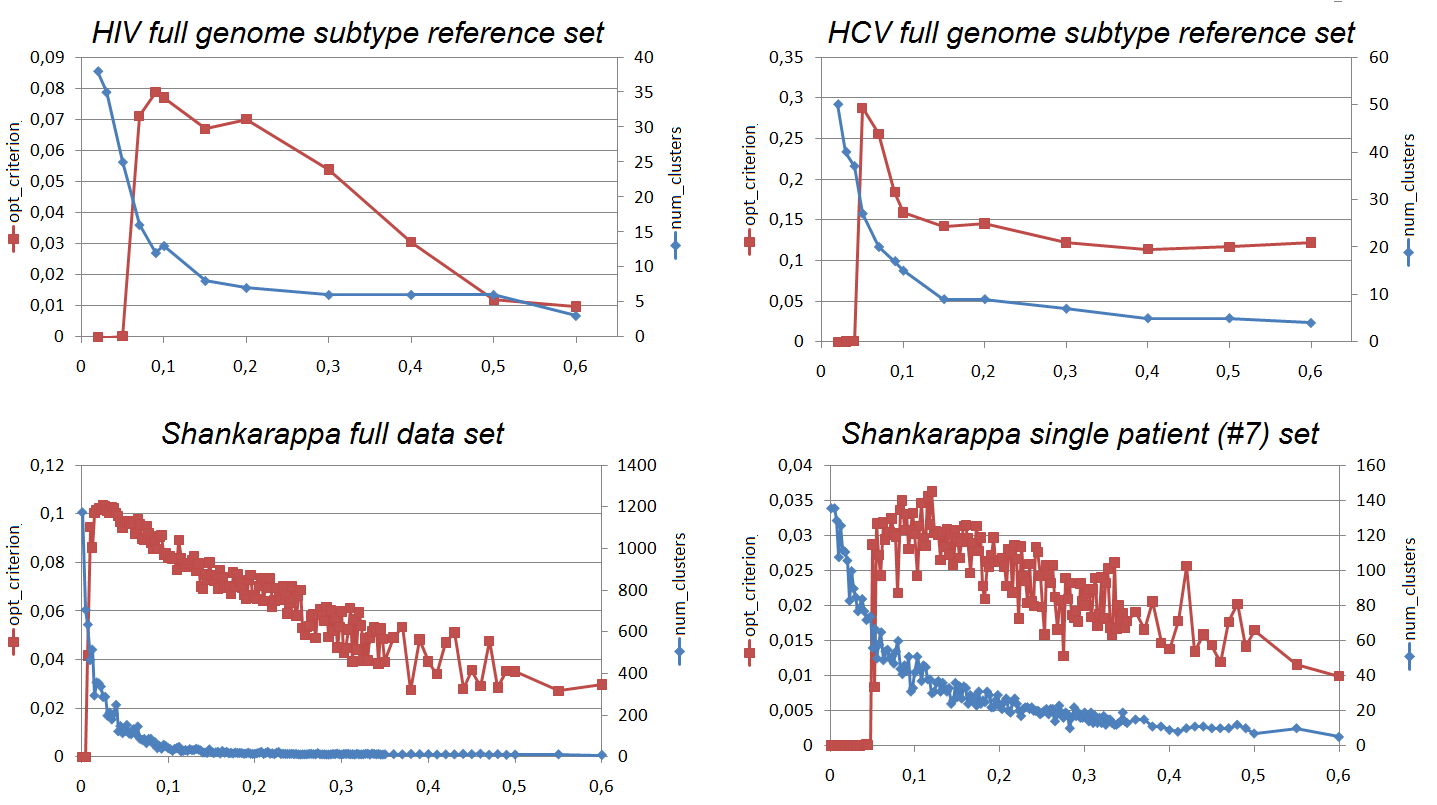

Supplement: Figure S2 — Optimisation of the threshold value by considering a cluster validity index (in this case maximising the difference between the median inter/intra-cluster distance distributions of a partition and the median values obtained from a random partition with the same number of clusters). (0.16 MB PNG) [file pone.0013619.s002.png]
